# Venous Stenting for Postthrombotic Iliocaval Venous Obstructive Disease: Clinical Efficacy and Mid-term Outcomes

**DOI:** 10.3400/avd.oa.22-00100

**Published:** 2022-12-25

**Authors:** Yuji Hoshino, Hiroyoshi Yokoi

**Affiliations:** 1 Section of Vascular Surgery, Fukuoka Sanno Hospital, Fukuoka, Fukuoka, Japan; 2 Section of Cardiovascular Medicine, Fukuoka Sanno Hospital, Fukuoka, Fukuoka, Japan

**Keywords:** postthrombotic syndrome, venous stenting, iliocaval venous obstruction, chronic venous insufficiency, venous ulcer

## Abstract

Postthrombotic syndrome (PTS) can cause both venous outflow obstruction and deep venous reflux, and results in severe symptoms of chronic venous disease. Venous stenting in the chronic iliocaval venous obstruction has been shown to be a safe and efficacious procedure. The long-term studies have shown the high patency rate, the good symptom relief, and the low recurrence rate of healed venous ulcerations. Although venous stenting has become a widely accepted treatment option in PTS with chronic venous occlusion or severe stenosis, it is not yet covered by insurance in Japan, and is being performed at limited facilities using off-label arterial stents. In this study, we performed venous stenting in 30 patients with the moderate and severe PTS. All patients showed significant improvement in their venous scores postoperatively, the Villalta score changed from a median of 16 before treatment to a median of 7 after treatment. Likewise, the Venous Clinical Severity Score and the Venous Disability Score dropped from a median of 13, 2.4 before treatment to 6, 1.2 after treatment, respectively. The primary patency and the secondary patency at 40 months were 93% and 96%, respectively. We report the excellent results and discuss current issues and future perspective in Japan. (This is secondary publication from J Jpn Coll Angiol 2021; 61: 99–105.)

## Introduction

Stenotic/obstructive lesions in the inferior vena cava (IVC)/iliac veins develop as a result of various etiologies. Regardless of the cause, the impaired venous outflow alone or combined with venous reflux in a region peripheral to the lesion can cause chronic venous insufficiency in lower limbs.

Known causes of the abovementioned stenotic/obstructive lesions include postthrombotic syndrome (PTS) and May–Thurner syndrome, which is also referred to as nonthrombotic iliac vein lesions (NIVL). PTS is the most common condition among these.

PTS is a complication that occurs in the chronic phase of deep vein thrombosis (DVT) and causes hypertension of veins in the lower limbs owing to the fact that organized residual thrombi cause venous stenosis/occlusion and valve reflux because of venous valve destruction. Clinical symptoms of PTS include swelling/heaviness in the lower limbs, developed/varicose superficial veins, venous claudication, stasis dermatitis/pigmentation, and ulceration. PTS is mild or moderate in most cases, and severe cases are relatively rare; however, severe cases accompanied by ulcers are intractable, and symptoms often recur repeatedly.

The severity of clinical symptoms tends to increase in PTS patients who present with occlusions at IVC/iliac vein levels combined with valve incompetence at or below the femoral vein level. Surgical revascularization procedures such as bilateral femoral vein bypass and bypass to IVC using synthetic graft have been used for the abovementioned obstructive lesions. However, outcomes of these surgical procedures have been unsatisfactory as the 4-year patency rate with saphenous vein grafts was 77% and the 1-year patency rate with synthetic grafts was 0%.^1)^ Recently, progress in endovascular treatment has substantially improved the treatment of venous diseases, and good treatment outcomes and long-term patency rates of iliac vein revascularization using stents have been reported.^2–5)^

In Japan, however, stent treatment of venous diseases has not been included in the national health insurance coverage and is currently conducted through the off-label use of arterial stents in a very limited number of institutions. Under such circumstances, we have performed venous stenting for PTS in 30 cases and for NIVL in 2 cases to date. Herein, we review these 30 cases of PTS and report treatment indications and results as well as future challenges associated with venous stenting.

## Subjects and Methods

Venous stents were placed in 31 limbs of 30 patients with PTS between December 2016 and December 2020. The patients included 15 women and 15 men with a mean age of 63 years (range, 31–91 years). In all cases, the etiology was PTS, and patients had a history of DVT. **Table 1** shows patient background, clinical stages, and details of hemodynamics. The clinical stages according to the Clinical, Etiology, Anatomy, and Pathophysiology (CEAP) classification system were C3 in 16% (5 limbs), C4a in 6% (2 limbs), C4b in 19% (6 limbs), C5 in 10% (3 limbs), and C6 in 49% (15 limbs). All patients were symptomatic. Although most of them had stasis dermatitis, all C3 patients with no skin lesions had venous claudication and pain in addition to swelling. Medians of preoperative Villalta score, Venous Clinical Severity Score (VCSS), and Venous Disability Score (VDS) were 16 (range, 6–26), 13 (range, 2–24), and 2.4 (range, 1–3), respectively.

**Table 1:** Table 1 Baseline demographics and clinical characteristics of 30 patients (31 limbs)

Median age, years	63 (31–91)	Anatomic lesions	
Female/male	15/15	Left side only	26 (87%)
Symptomatic	100%	Right side only	3 (10%)
Bilateral symptoms	1 (3%)	Bilateral+IVC	1 (3%)
CEAP class		CIV only	3 (10%)
C3	5 (16%)	CIV/EIV	12 (40%)
C4a	2 (6%)	CIV/EIV/CFV	14 (47%)
C4b	6 (19%)	Degree of obstruction	
C5	3 (10%)	Occlusion	26 (84%)
C6	15 (49%)	Non-occlusive	5 (16%)
Median Villalta score	16 (6–26)	Inspilateral FV	
Median VCSS	13 (2–24)	Reflux	10 (32%)
Median VDS	2.4 (1–3)	Occlusion	5 (16%)
Previous surgery (total)	12 (39%)		
Superficial surgery	5 (16%)		
Perforator surgery	3 (10%)		
Superficial+perforator surgery	4 (13%)		

Continuous variables are reported as median (range). Categorial variables are reported as number (%).CEAP: Clinical, Etiology, Anatomy, and Pathophysiology; VCSS: venous clinical severity score; VDS: venous disability score; IVC: inferior vena cava; CIV: common iliac vein; EIV: external iliac vein; CFV: common femoral vein; FV: femoral vein

**Figure 1:**
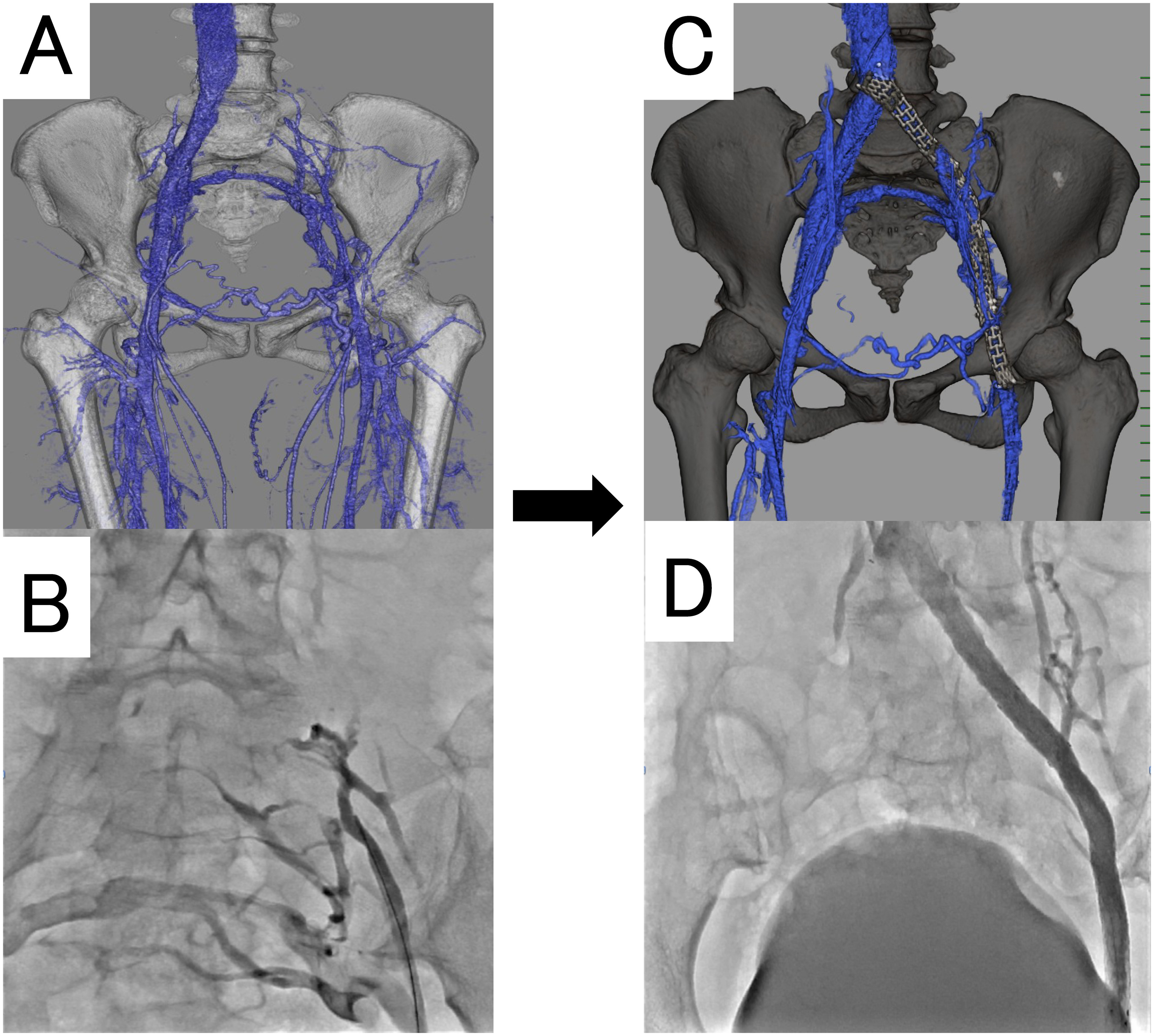
Fig. 1 Postthrombotic iliac vein occlusion and the venous stenting. (**A**) Contrast-enhanced computed tomography (CT) ﬁnding: before stenting. CT scan shows a typical thrombotic iliac vein occlusion with transpelvic collaterals. (**B**) Ascending venography ﬁndings: before stenting. Transfemoral venogram shows a typical thrombotic iliac vein occlusion with transpelvic collaterals. (**C**) Contrast-enhanced CT ﬁnding: after stenting. CT scan after stenting shows no stenosis or collaterals. (**D**) Ascending venography ﬁndings: after stenting. The stenting is carried into the left common iliac vein, external iliac vein, and common femoral vein.

In all patients, preoperative color Doppler ultrasonography and computed tomography (CT) revealed obstruction or ≥80% stenosis in the IVC/iliac vein region (**Fig. 1A**). Lesions were identified on the left side in 26 cases (87%), on the right side in 3 cases (10%), and on both sides in 1 case (3%).

Three limbs (10%) had solitary lesions of the common iliac vein (CIV); 12 limbs (40%) had CIV/external iliac vein (EIV) lesions; 14 limbs (47%) had CIV/EIV/common femoral vein (CFV) lesions; and 1 case had a lesion encompassing the whole region from the IVC to CFV (3%). Total obstruction was found in 26 limbs (84%).

Kistner grade ≥3 reflux in the femoral vein (FV) was found in 10 limbs (32%), and FV obstruction was found in 5 limbs (16%).

Of 30 patients, 5 (16%) had a history of surgery for varicose veins, 3 (10%) had a history of surgery for incompetent perforator veins, and 4 (13%) had undergone both. In all cases, venous stasis was refractory as the condition persisted while patients received treatment for concomitant superficial venous insufficiency or perforator vein incompetence and were undergoing compression therapy, including the use of elastic stockings.

Percutaneous venous access was from the ipsilateral FV, ipsilateral CFV, ipsilateral great saphenous vein, or right internal jugular vein; a bidirectional approach from the right internal jugular vein and ipsilateral leg vein (common femoral/femoral/great saphenous vein) was used when the affected vein was obstructed completely, and a unidirectional approach did not allow for passage of the guide wire (**Table 2**). After the venous access was established, unfractionated heparin was administered intravenously, the iliac vein was imaged with a contrast agent via a 9Fr sheath, and a 0.035, 0.018, or 0.014-inch guide wire was allowed to penetrate the occlusion/severe stenosis site before an intravascular ultrasound (IVUS) examination.

**Table 2:** Table 2 Procedural characteristics of 31 stented limbs

Access vessel	
FV	10 (32%)
CFV	5 (16%)
Great saphenous vein	2 (6%)
Internal jugular vein	3 (10%)
Bidirectional^a^	11 (36%)
Stent location	
Isolated CIV	3 (10%)
CIV/EIV	12 (40%)
CIV/EIV/CFV	14 (47%)
IVC/CIV/EIV/CFV	1 (3%)
Mean stent number	2.3 (1–5)
Mean Stent diameter, mm	
IVC	14×2
CIV	13.9 (12–16)
EIV	12.3 (10–14)
CFV	10.1 (10–12)
Lesion extending into CFV	16 (51%)
Lesion extending into IVC	2 (6%)

Continuous variables are reported as median (range). Categorial variables are reported as number (%). ^a^Internal jugular vein and CFV/FV/great saphenous vein. IVC: inferior vena cava; CIV: common iliac vein; EIV: external iliac vein; CFV: common femoral vein; FV: femoral vein

A self-expanding stent was placed at each obstruction/severe stenosis site after stepwise pre-dilation with a balloon dilatation catheter of a diameter nearly equal to the stent (**Figs. 1C** and **1D**). Of a total of 71 stents used for 31 limbs of 30 patients, 69 (97%) were arterial stents such as SMART (Cordis Corporation, Bridgewater, NJ, USA), Luminexx (Bard Peripheral Vascular, Tempe, AZ, USA), Epic (Boston Scientific Corporation, Natick, MA, USA), and Wallstent (Boston Scientific Corporation, Natick, MA, USA), and only two (3%) were venous stents (ZilverVena; Cook Medical, Bloomington, IN, USA). As a guide of stent diameter, a 14-mm stent was used for the CIV; a 12-mm stent was used for the EIV; a 10-mm was used for the CFV; and two 14-mm stents were used for double-barrel stenting in the IVC from the left and right CIVs (**Table 2**). As to the stent length, one or more stents were selected from among 40-, 60-, 80-, and 100-mm stents based on the length of each lesion; when multiple stents were used, they were placed to entirely cover the length of the lesion with an approximately 10-mm overlap.

After post-stenting balloon dilation, IVUS and angiography were used to confirm revascularization. From the perioperative to the postoperative periods, patients used elastic compression garments and foot pumps in combination before discharge and continued using elastic compression garments after discharge.

Patients underwent double therapy with an antiplatelet agent and an anticoagulant for 6 months after stenting. After patency at the stent site was confirmed at the follow-up visit 6 months after stenting, patients continued monotherapy with an anticoagulant.

The stent patency was evaluated through color Doppler ultrasonography or contrast-enhanced CT the day after, 1 month, and 3 months after surgery and every 6 months thereafter (**Fig. 1C**). Furthermore, in-stent restenosis was defined as ≥50% stenosis on images. Improvements in clinical symptoms were evaluated using the Villalta score, VCSS, and VDS 1 month after stenting.

The Kaplan–Meier survival analysis was used to determine the cumulative stent patency rates. Primary patency was defined as the absence of restenosis or occlusion in the treated vessel without revascularization to maintain the patency; assisted primary patency as no restenosis or occlusion in the treated vessel with revascularization to maintain the patency; and secondary patency as patency re-established through revascularization after total occlusion of the treated vessel. The t-test was used to compare pre- and post-treatment clinical condition scores between the two groups, and P<0.05 indicated statistical significance.

Examinations and treatment procedures related to this article were conducted with approval from the Institutional Review Board (approval numbers: 20-FS-467, 21-FS-482) and adequate informed consent.

## Results

**Table 2** shows items related to endovascular treatment. Percutaneous venous access was established from the ipsilateral FV in 10 limbs (32%), the ipsilateral CFV in 5 limbs (16%), the ipsilateral great saphenous vein in 2 limbs (6%), and the right internal jugular vein in 3 limbs (10%). Of 26 limbs (84%) with total occlusion (**Table 1**), 11 (36%) required a bidirectional approach because a unidirectional approach did not allow for passage of the guide wire. The stents were placed in the CIV alone in 3 cases (10%), from the CIV to the EIV in 12 cases (40%), from the CIV to the CFV in 14 cases (47%), and over a whole region from the IVC to the CFV in 1 case (3%). In most cases, the stents were placed in the iliac vein; however, stent extension to the ipsilateral CFV was required in 51% of all cases. The mean diameters of stents used in the CIV, EIV, and CFV were 13.9 mm (range, 12–16), 12.3 mm (range, 10–14), and 10.1 mm (range, 10–12), respectively; the mean number of stents used per case was 2.3 (range, 1–5).

**Figure 2:**
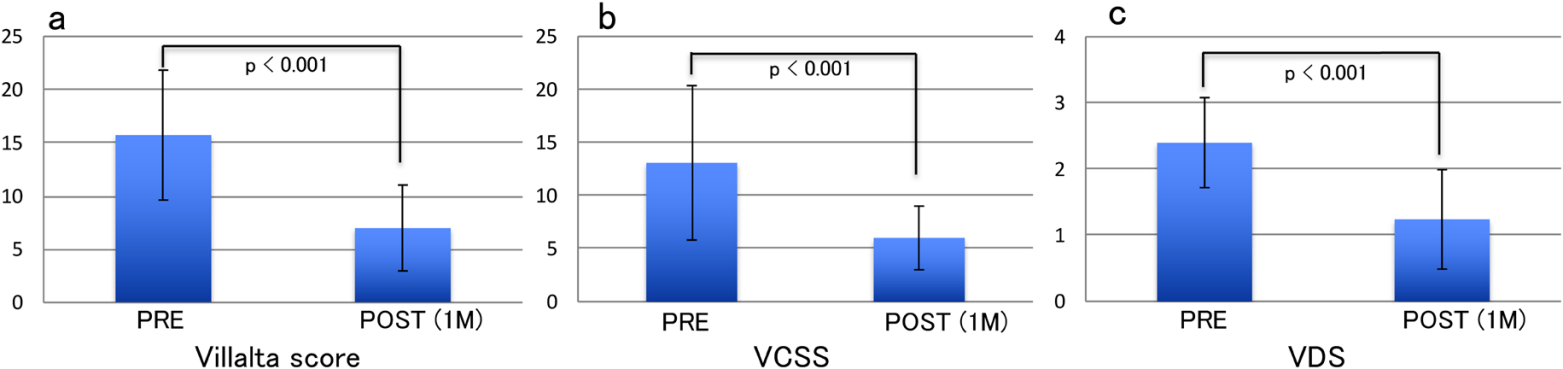
Fig. 2 Change of clinical scores before and after the intervention. (**a**) Villalta score, (**b**) VCSS: venous clinical severity score, (**c**) VDS: venous disability score; M: Months.

No patients experienced complications including death and pulmonary embolism during the perioperative period and the follow-up period (median, 28 months; range, 2–48 months). All 30 patients showed clinical improvements; the median Villalta score improved to 7 (range, 1–16) after endovascular treatment from 16 before treatment (P<0.001), and the median VCSS and VDS decreased to 6 (range, 1–13) and 1.2 (range, 0–2) after endovascular treatment from 13 and 2.4 before treatment, respectively (P<0.001 for both) (**Table 3**, **Fig. 2**). In all cases with ulcers, the ulcers epithelialized and were cured within 1 month after stenting.

**Table 3:** Table 3 Results: all follow-up assessments in 30 stented limbs

Follow-up, month	28 (2–48)
Re-thrombosis	1 (3%)
Restenosis	4 (13%)
Primary patency	93% at 40 M
Assisted-primary patency	96% at 40 M
Secondary patency	96% at 40 M
Median Villalta score (1 M)	7 (1–16)
Median VCSS (1 M)	6 (1–13)
Median VDS (1 M)	1.2 (0–2)

Continuous variables are reported as median (range). Categorial variables are reported as number (%).VCSS: venous clinical severity score; VDS: venous disability score; M: Months

**Figure 3:**
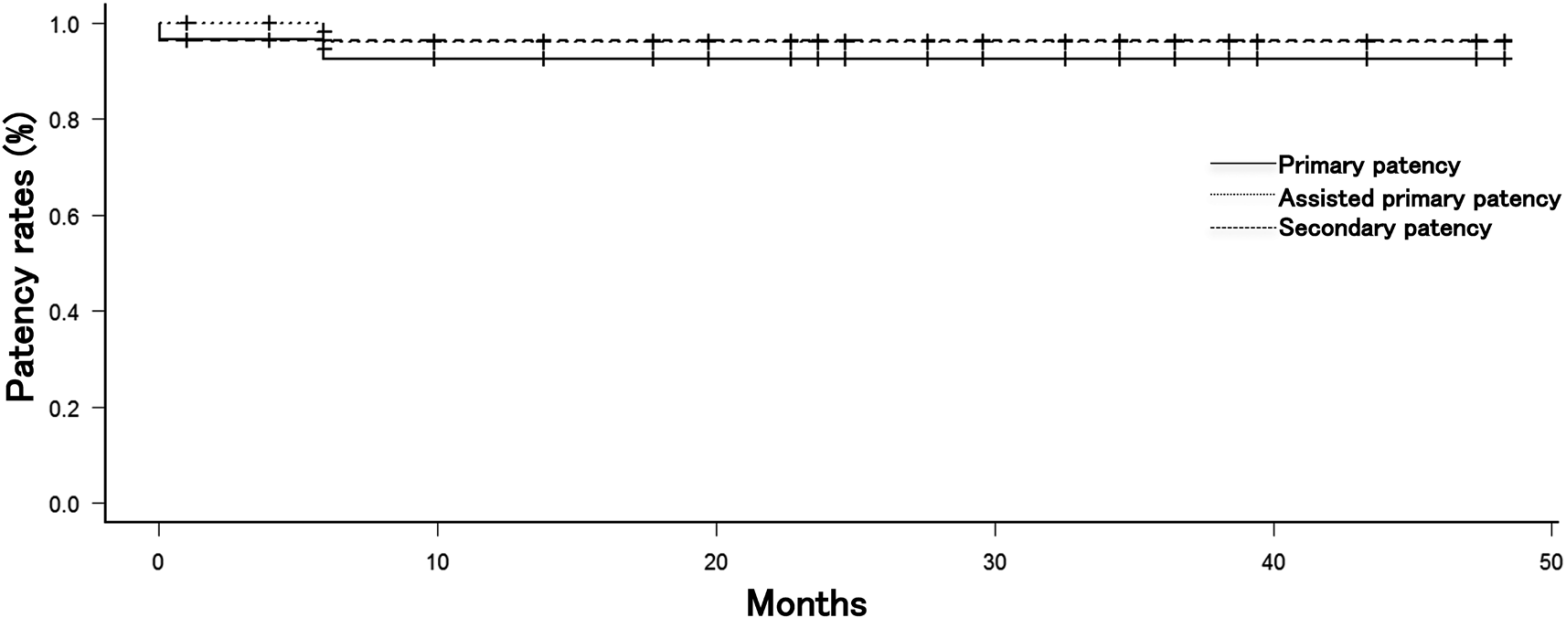
Fig. 3 Cumulative primary, assisted-primary, and secondary patency rates of 31 limbs after venous stenting.

Stent thromboembolism occurred during the follow-up period in 1 case (3%). In this case, the stents were obstructed acutely the day after placement, and recanalization was achieved through thrombolytic therapy and placement of additional stents on the same day. Stent restenosis occurred in 4 cases (13%); among these cases, stent restenosis was found in the assessment 1 month after surgery in 3 cases and in the assessment 6 months after surgery in 1 case. In all of these cases, patients remained asymptomatic until restenosis was found in a scheduled follow-up examination. In one of these cases, an additional stent was placed. Long-term stent patency until the final follow-up was achieved in all cases, including the abovementioned 2 cases in which additional stents were placed and 3 cases in which stenosis was noted but monitored via conservative follow-ups. The primary, assisted-primary, and secondary patency rates at 40 months were 93%, 96%, and 96%, respectively (**Table 3**, **Fig. 3**).

## Discussion

Previously, venous stents have been reported to ensure good patency rates and outcomes.^2–5)^ In the largest cohort study following 464 limbs of PTS patients for 5 years after venous stenting, Neglén et al.^2)^ have reported an ulcer healing rate of 55% and significant improvements in clinical symptoms, indicating a high treatment success rate and good treatment outcomes. Guidelines of the Society for Vascular Surgery and the American Venous Forum also propose endovascular treatment as the first choice for chronic obstructive venous diseases with skin lesions.^6)^

Appropriate use criteria (AUC) reported in the Journal of Vascular Surgery in 2020 provided clearer criteria for iliac vein/IVC stenting. Specifically, stenting is the first-choice treatment when (1) superficial venous reflux is absent; (2) patients are symptomatic and fall into CEAP classes C4–C6 with higher levels of clinical severity; and (3) IVUS reveals ≥50% stenosis/occlusion.^7)^ Of patients treated in our hospital, 95% were accounted for by PTS patients with skin lesions who fell into ≥C4 and did not have superficial venous insufficiency or perforator vein incompetence; thus, these patients were considered “appropriate” according to the above criteria. Currently, venous stenting is not covered by the national health insurance system in Japan and is performed through the off-label use of arterial stents. Thus, we placed the highest priority on strict adherence to the above criteria. Therefore, deep vein incompetence was the main cause in the vast majority of cases, although the number of cases was not large. As a result, treatment outcomes were very good. Of the 30 patients who underwent stenting in this study, 12 had undergone some surgery for varicose veins of the lower extremities, accounting for a relatively large proportion (39%). This suggested that cases of varicose veins of the lower extremities in which symptoms are exacerbated after usual treatment, include many cases of deep vein incompetence.

Most lesions indicated for stenting were in the iliac vein region; however, stents had to be placed in an extensive region to CFV across the inguinal ligament in 51% of the cases. For venous diseases, a previous report has stated that stent placement in the CFV peripheral to the inguinal ligament is not problematic^8)^; however, many other studies have shown poor treatment outcomes in cases in which stents were placed across the inguinal ligament.^2,9)^ Currently, stent placement across the inguinal ligament is considered to have no safety problems, although pros and cons remain controversial. However, treatment results tend to become poorer when the etiology is PTS and obstructive/stenotic lesions are also found in the CFV peripheral to the inguinal ligament because the risk for stent occlusion increases.^10)^ In the only case of stent occlusion in this study, the stents were obstructed acutely the day after placement. The original lesion was a total occlusion over a long segment from the left CIV to the CFV, and the stents had to cover a segment peripheral to the inguinal ligament. This patient achieved successful revascularization through thrombolytic therapy on an emergency basis and placement of additional stents in a further peripheral segment, i.e., not covering the deep femoral veins, on the day the stent occlusion was found. Thereafter, patency was maintained for the long term. The importance of inflow to achieve long-term patency has previously been pointed out.^10)^ In 5 cases in which stenotic/obstructive lesions were also found in the FV cases in this study, the deep femoral and great saphenous veins were important inflows.

Stent restenosis was found in 4 cases (13%) in this study. In all of these four cases, stent restenosis was found via ultrasonography in scheduled postoperative follow-up examinations, whereas patients were clinically asymptomatic. In one of these cases in which the patient underwent placement of additional stents, the original lesion was a total occlusion of the left CIV, and stents were placed from the left CIV to the EIV. Six months after stenting, ≥50% stenosis was noted in the stented site of the EIV. Since this was the first case of postoperative restenosis encountered in our hospital, additional stents were placed to cover the stenotic lesion and its peripheral side. In the other 3 cases, the original lesions were total occlusions from the left CIV to the EIV, and stent restenosis was found within 1 month after placement. In these cases, it was difficult to determine whether stenosis was caused by fresh thrombi or intimal thickening and whether additional stents were really necessary. Therefore, evaluation via direct observation with an angioscope was conducted in one of these cases. The result revealed the absence of intrastent thrombi, suggesting that stent stenosis was caused via intimal thickening. Furthermore, this examination confirmed that the intravascular space at the stenosis site remained relatively intact. Thus, these patients were followed up with no additional procedures. Fortunately, aggravation of stenosis or stent occlusion was not noted in the subsequent regular follow-up examinations in these cases or other cases. Although the number of cases was too small to make a definitive conclusion, these findings suggest that stent restenosis was not caused by fresh thrombi. Neglén et al. have also reported that attempts of thrombolytic therapy in patients with early stent restenosis were ineffective.^9)^ As to the necessity of an additional therapeutic intervention, placement of additional stents is considered unnecessary for asymptomatic patients, based on the current AUC.

As oral medications after stenting, anticoagulants are considered appropriate when the etiology is PTS, while antiplatelet agents are considered appropriate when the etiology is NIVL.^8)^ However, no clear evidence on this matter, including the treatment duration, has been shown. Our treatment protocol is to use double therapy with an antiplatelet agent and an anticoagulant for the first 6 months after stenting because the anticoagulant effect is also required in an early phase until struts are covered with the endothelium to some extent, switch to monotherapy with an anticoagulant once the stent patency is confirmed in the sixth month, and then continue the anticoagulant monotherapy.

Fortunately, long-term stent patency was successfully achieved and no complications such as bleeding occurred in all cases of venous stenting performed at our hospital.

Optimal stent sizes for venous stenting are generally large; 14–16 mm for CIV, 12–14 mm for EIV, and 10–12 mm for CFV.^8)^ All venous stents used in the cases reported here were generally in line with the abovementioned sizes. In one case, however, a thinner stent (10 mm diameter) had previously been placed in the left CIV at a different hospital.^11)^ A new severe stenotic lesion subsequently developed in the peripheral EIV, and stasis dermatitis relapsed. As treatment, a thicker 12-mm stent was additionally placed in the EIV peripheral to the 10-mm CIV stent. Fortunately, the treatment was successful, and dermatitis was also cured. Nevertheless, the case reminded us of the necessity of clear treatment guidelines on venous stenting in Japan. It is also desirable to make wider stents and venous stents used in other countries available in Japan. In most of the cases included in the present study, venous stenting was performed through the off-label use of arterial stents. However, treatment outcomes were satisfactory thus far. It is desirable that stents dedicated for veins become available in Japan and that the clinical data in Japan can adequately be compared with those obtained in other countries.

## Conclusion

These findings suggest the high effectiveness of venous stenting for treatment of severe PTS with stenotic/obstructive lesions in the iliac veins and the IVC. Decision-making about treatment indication is critical for appropriate treatment. Clear guidelines for proper use are also needed in Japan.

## Notes

This is secondary publication from J Jpn Coll Angiol 2021; 61: 99–105.
